# Short-wave infrared cavity resonances in a single GeSn nanowire

**DOI:** 10.1038/s41467-023-40140-0

**Published:** 2023-07-20

**Authors:** Youngmin Kim, Simone Assali, Hyo-Jun Joo, Sebastian Koelling, Melvina Chen, Lu Luo, Xuncheng Shi, Daniel Burt, Zoran Ikonic, Donguk Nam, Oussama Moutanabbir

**Affiliations:** 1 School of Electrical and Electronic Engineering, Nanyang Technological University, 50 Nanyang Avenue, Singapore, 639798 Singapore; 2 Department of Engineering Physics, École Polytechnique de Montréal, C.P. 6079, Succ. Centre-Ville, Montréal, QC H3C 3A7 Canada; 3 School of Electronic and Electrical Engineering, University of Leeds, Leeds, LS2 9JT UK

**Keywords:** Nanowires, Nanowires

## Abstract

Nanowires are promising platforms for realizing ultra-compact light sources for photonic integrated circuits. In contrast to impressive progress on light confinement and stimulated emission in III-V and II-VI semiconductor nanowires, there has been no experimental demonstration showing the potential to achieve strong cavity effects in a bottom-up grown single group-IV nanowire, which is a prerequisite for realizing silicon-compatible infrared nanolasers. Herein, we address this limitation and present an experimental observation of cavity-enhanced strong photoluminescence from a single Ge/GeSn core/shell nanowire. A sufficiently large Sn content ( ~ 10 at%) in the GeSn shell leads to a direct bandgap gain medium, allowing a strong reduction in material loss upon optical pumping. Efficient optical confinement in a single nanowire enables many round trips of emitted photons between two facets of a nanowire, achieving a narrow width of 3.3 nm. Our demonstration opens new possibilities for ultrasmall on-chip light sources towards realizing photonic-integrated circuits in the underexplored range of short-wave infrared (SWIR).

## Abstract (web summary)

Group-IV nanowires hold great promise for building ideal light sources for photonic integrated circuits. This study presents an observation of cavity resonances in a single GeSn nanowire, laying the foundation for realizing group-IV nanowire lasers.

## Introduction

Nanowires grown by bottom-up synthesis hold great promise for building ultrasmall and power-efficient light sources for photonic integrated circuits (PICs)^1^. In the past two decades, several research groups reported various types of nanowire light sources and lasers using a variety of III–V and II–VI semiconductor materials such as GaAs, InAs, and CdS^2–9^. Recently, CMOS-compatible group-IV materials such as Ge and GeSn have attracted much attention as a gain medium for on-chip lasers operating at wavelengths deep in the infrared relevant to free-space communication and sensing applications^10–15^. Particularly, group-IV bottom-up nanowires have the potential to produce the smallest, CMOS-compatible on-chip light sources, thus motivating many researchers to grow high-quality GeSn^16–24^. In the last decade, the photoluminescence characteristics of GeSn nanowires have been unveiled^16–22,25^. Temperature-dependent photoluminescence from an ensemble of vertically grown nanowires revealed the direct bandgap nature of GeSn nanowires with high Sn contents up to ~19 at%^18,19^. However, possibly due to the mode leaking from nanowires into the substrate and because of the inability to precisely measure the emission characteristic of a single nanowire, it has thus far remained elusive whether it is feasible to achieve a strong cavity resonance in a single group-IV nanowire. A very recent study reported clear photoluminescence emission from a single Ge/GeSn core/shell nanowire transferred onto an Au and Si substrate, signs of cavity resonances were not detected^22^.

Herein, we present an experimental observation of cavity resonances from a single Ge/GeSn core/shell nanowire with a uniform Sn content of ~10 at%. The use of a very thin Ge core (20 nm and smaller) is a critical approach to enhance the elastic strain relaxation in the nanowire radial heterostructure, prevent growth-extended defects, and minimize the gradient in Sn content thus resulting in a uniform composition across the ~500 nm-thick Ge/Ge_0.90_Sn_0.10_ nanowire and along its entire length of several micrometers. Strongly enhanced photoluminescence at low temperatures indicates the realization of direct bandgap GeSn light-emitting media. In stark contrast to a broadband spontaneous emission characteristic from as-grown vertical nanowires, a single nanowire transferred onto a SiO_2_ layer on a Si substrate shows cavity resonances that are in excellent agreement with finite-difference time-domain (FDTD) simulations. The linewidths of the cavity resonances initially reduce significantly with increasing pump power due to the increased material gain, but the resonant peaks broaden rapidly and disappear at higher pump powers. Theoretical calculations based on **k**⋅**p** method combined with numerical analyses indicate that GeSn nanowire reaches the transparency condition very closely, but the material loss dominates the material gain at increased pump powers, hindering the observation of lasing. Via FDTD simulations and theoretical calculations, we propose a viable pathway toward achieving lasing by reducing the optical losses in a cavity via a simple lithography step. This experimental demonstration provides a strong motivation for delving into group-IV nanowire light emitters to achieve on-chip CMOS-compatible nanowire SWIR lasers.

## Results

### Nanowires growth and characterization

**Fig. 1:**
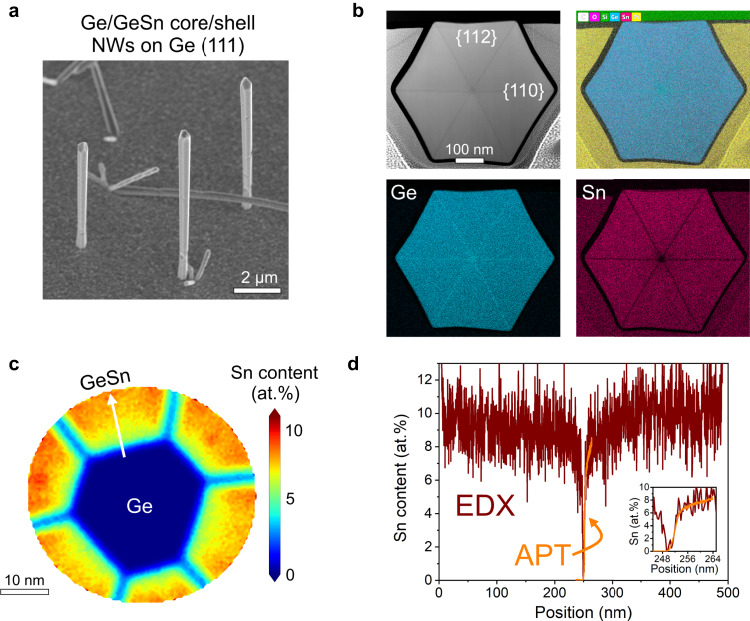
Nanowire structure and composition. **a** SEM image of Ge/Ge_0.90_Sn_0.10_ core/shell nanowires. **b** Cross-sectional HAADF-STEM image and compositional EDX maps showing C, O, Si, Ge, Sn, Pt overlayed as well as the Ge and Sn map. **c** APT measurement showing the Ge core and the inner portion of the Ge_0.90_Sn_0.10_ shell. The line profile (white arrow) is shown in **d**. **d** Plot of the Sn content as a function of the distance along the radial direction of the shell estimated from APT and EDX.

The Ge/Ge_0.90_Sn_0.10_ core/shell nanowires were grown using 20 nm Au colloids dispersed on a Ge(111) substrate using a chemical vapor deposition (CVD) reactor (see methods for more details). The scanning electron micrograph (SEM) in Fig. 1a shows as-grown Ge/Ge_0.90_Sn_0.10_ core/shell nanowires at lengths exceeding 10 μm and diameters larger than 400 nm. A small inverse tapering is visible, as a result of the enhanced precursor decomposition in the proximity of the Au droplet^18^. Scanning Transmission Electron Microscopy (STEM) and Atom Probe Tomography (APT) measurements were performed to unveil the structural properties of the nanowire heterostructure. Cross-sectional high-angle annular dark field (HAADF) STEM images and energy-dispersive X-ray spectroscopy (EDX) compositional maps are shown in Fig. 1b. The typical sunburst-like morphology of the Ge_0.90_Sn_0.10_ shell, with Sn-rich {112} wide facets and Ge-rich {110} narrow facets^18,26^, is preserved across the entire 250 nm thickness of the shell. The composition of the GeSn shell was characterized using APT measurements, as displayed in Fig. 1c. Since it is challenging to perform APT measurements on nanowires with diameters larger than 200 nm, a second nanowire sample was grown with identical conditions and a thinner ~30 nm shell. The compositional profile estimated along the <112> radial direction of the shell from EDX and APT measurements are plotted in Fig. 1d. Due to the limited field-of-view of the APT instrument only ~60 % of the cross-section of the nanowires is visible, however, the composition of the group-IV nanowires can be reliably measured^27,28^. The EDX curve, which is affected by a background signal, was vertically translated to overlap with the APT curve. A steep transition (~5 nm-wide) to ~6 at% Sn is visible along the <112> direction, while the incorporation of Sn gradually increases toward the outer region of the shell, eventually reaching a uniform Sn~10 at% at more than 75 nm away from the Ge core. Along the <110> direction of the shell a rather constant composition of Sn~3 at% was obtained. We note that the nanowires offer excellent strain minimization due to their one-dimensional geometry (i.e. highly compliant substrate), and the residual compressive strain is progressively reduced with increasing shell thickness^29^, until an equilibrium configuration with higher content uniformity is reached. Earlier studies on Ge/GeSn core/shell nanowire heterostructures employed Ge cores with diameters of 50 nm or larger, which resulted in significant compositional fluctuations and limited the thickness of defect-free shells to less than 150 nm. In the present work, by reducing the Ge core down to 20 nm the transition to 10 at% is reached within 50 nm from the interface with Ge, thus allowing the remaining thickness (~200 nm) of the shell to maintain a uniform composition (See Supplementary Fig. 4 for more details). The ability to obtain a highly uniform Sn content in GeSn nanowires while limiting the compressive strain is of paramount importance in enabling cavity modes in the nanowires. Additionally, reducing the diameter of the Ge core enhances strain relaxation and thus minimizes the extended defects. The latter hinders realizing the cavity mode by acting as trap states, causing non-radiative recombination that can degrade light emission efficiency in the materials^30^. Hence, it is crucial to minimize defects in materials to enable optical emission at room temperature. Early studies hinted at the detrimental effect of structural defects and inhomogeneous Sn incorporation in the GeSn shell on the optical properties of nanowires. For example, Ge/GeSn core/shell nanowires grown using a 50 nm diameter Ge core exhibit optical emission at 300 K^18^, while the emission vanishes ~100 K for nanowires with 100 nm diameter Ge core^21^. This behavior is associated with the improved structural quality of the GeSn nanowire when the Ge core diameter is reduced from 100 nm to 50 nm. These prior studies highlight the importance of using smaller core in minimizing defects in GeSn for achieving good optical properties.

### Direct bandgap emission from as-grown Ge/GeSn core/shell nanowires

**Fig. 2:**
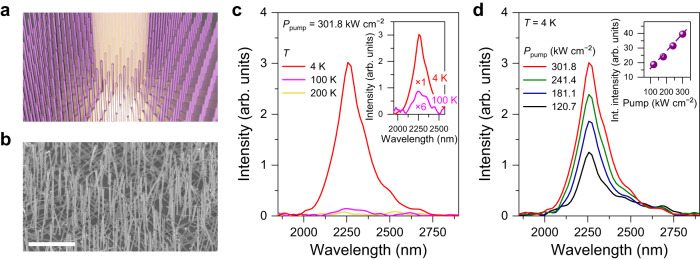
Direct bandgap emission from as-grown Ge/GeSn core/shell nanowires. **a** Schematic illustration of the photoluminescence measurements on the as-grown nanowire sample, showing several nanowires being optically pumped simultaneously. **b** Tilted-view SEM image of an as-grown nanowire sample. Scale bar, 10 µm. Vertically standing as-grown nanowires are densely distributed on a Ge layer with a spacing of 1–5 μm. It is not feasible to study the optical emission characteristics of a single nanowire since the beam diameter of the optical pump in our setup is ~10 μm. **c** Temperature-dependent photoluminescence spectra of as-grown nanowires at temperatures of 4 K (red), 100 K (pink), and 200 K (yellow) under a pump power density of 301.8 kW cm^−2^. The emission intensity rapidly reduces at higher temperatures, indicating that the GeSn light-emitting medium is a direct bandgap material. Inset: magnified spectra of 4 K and 100 K. The 100 K spectrum is multiplied by a factor of 6 for clarity. **d** Power-dependent photoluminescence spectra of as-grown nanowires taken at 4 K, without showing any evidence of cavity resonances. The peak position of the spectra stays at ~2275 nm as the pump power is increased, indicating a negligible pump-induced heating effect in the nanowires. Inset: integrated emission intensity that increases linearly as a function of the pump power, further confirming the absence of the heating effect.

To study whether the bandgap of the light-emitting medium in the nanowires is intrinsically direct or indirect, we conducted temperature-dependent photoluminescence measurements on as-grown nanowires (see Methods for the detailed description of photoluminescence spectroscopy). Fig. 2a shows a schematic illustration of our experiments on the as-grown nanowire sample, showing several nanowires being optically excited simultaneously. The schematic here is to illustrate that there exist many nanowires to be simultaneously pumped since the optical pumping area is large even with a tightly focused pump laser spot. It should be noted that the experimentally grown nanowire array (Fig. 2b) is not as perfectly periodic and vertical as in the schematic. Fig. 2b presents the SEM image of the as-grown nanowire sample. It is evident from the SEM image that vertically standing as-grown nanowires are densely packed on a Ge layer with distances between nanowires of 1–5 μm. Since the diameter of the optical pump in our setup is ~10 µm, a few nanowires are excited during the measurements. Fig. 2c shows the direct bandgap emission spectra from the as-grown nanowire sample at 200 K (yellow), 100 K (pink), and 4 K (red). A fixed pump power density of 301.8 kW cm^−2^ was used at all temperatures. The inset to Fig. 2c compares two spectra measured at 4 K (red) and 100 K (pink). The spectrum for 100 K is multiplied by a factor of 6 for clarity. A broadband spontaneous emission peak at ~2275 nm was clearly observed at 4 K. The measured peak position is consistent with other reports using GeSn with a similar Sn content^13,31^. It is worth noting that emission intensity significantly increases at lower temperatures, which is typical behavior of direct bandgap materials^32^. At lower temperatures, the carrier density in the lowest direct Γ-valley in a direct bandgap material can be drastically increased^33^, resulting in stronger photoluminescence. Also, non-radiative recombination processes can be suppressed at lower temperatures, further increasing the emission intensity^11^. To further confirm the direct bandgap nature of the GeSn nanowire, we compared the experimentally measured emission energies against theoretically calculated bandgap energies. Supplementary Fig. 6 presents calculated bandgap energies for Γ- and L-valleys as a function of the Sn content, showing that the indirect-to-direct cross-over occurs at a Sn content of ~8 at%. The Sn content of 10 at% in the nanowire, which is an experimentally measured value using APT, is beyond this indirect/direct cross-over point. At this Sn content of 10 at%, the calculated bandgap energy for the Γ valley is 0.5505 eV, which corresponds to a wavelength of 2255 nm, which is in excellent agreement with the experimentally measured emission peak of 2251 nm (highlighted in an orange circle in Supplementary Fig. 6). This theoretical benchmarking further confirms the direct bandgap nature of GeSn nanowire in addition to the temperature-dependent photoluminescence characteristics. Fig 2d presents the emission spectra from the as-grown nanowire sample at different pump power densities at 4 K. The emission peak position remains the same as the pump power density is increased, confirming the negligible pump-induced heating effect in the sample at all pump power levels used in this study. The inset to Fig. 2d shows that the integrated emission intensity linearly increases as a function of pump power density, further confirming the absence of any heating effect. Unlike other reports showing strong cavity effects in vertically standing III-V and II-VI nanowires^2,34,35^, we were unable to observe any cavity modes in as-grown samples. From our FDTD simulations, we confirmed that the small refractive index contrast between the nanowires and the Ge substrate causes a significant leakage of optical fields from the nanowires to the substrate (see Supplementary Note 3 for more details on the FDTD simulations). We would like to emphasize that even if the optical mode can be well confined in the nanowires, it remains challenging to probe the potential to confine light in a single nanowire because a relatively large pump beam spot size prohibits studying the emission characteristics of a single nanowire, as shown in Fig. 2a.

### Cavity resonances from a single Ge/GeSn core/shell nanowire

**Fig. 3:**
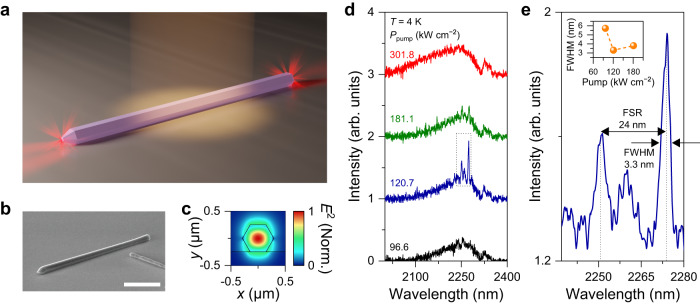
Cavity resonances from a single Ge/GeSn core/shell nanowire. **a** Schematic illustration of our photoluminescence measurements on the transferred nanowire sample, showing only a single nanowire being optically excited. **b** Tilted-view SEM image of a single nanowire transferred onto an SiO_2_ layer on an Si substrate. Scale bar, 7 μm. It is feasible to pump only a single nanowire within the pumping beam spot of ~10 µm in our setup. **c** Simulated cross-sectional optical mode profile of the transferred nanowire with a diameter of 500 nm at the wavelength of 2275 nm showing that the optical field can be strongly confined in a nanowire. **d** Power-dependent photoluminescence spectra from a single transferred nanowire at 4 K. The spectra show the onset (black), strong enhancement (blue), weakening (green), and dissipation (red) of the cavity resonances as the pump power is increased. **e** Magnified view of a spectrum taken at 120.7 kW cm^−2^ in a black dashed line in Fig. 3a, showing two cavity resonances with an FSR of ~24 nm and narrow FWHM of ~3.3 nm at 2275 nm. Inset: The linewidth change of the cavity mode at 2275 nm as a function of pump power. The linewidth narrows from ~5.8 to ~3.3 nm and broadens to ~3.8 nm as the pump power is increased.

To investigate the possibility of confining light in a single GeSn nanowire, we transferred as-grown nanowires onto an SiO_2_ layer on an Si substrate (see Supplementary Note 1 and 2 for more details on the procedure to transfer the nanowires) and performed photoluminescence measurements on the transferred nanowires (See Methods for the detailed description on photoluminescence spectroscopy). Fig. 3a shows a schematic illustration of these experiments. It is worth noting that it is possible to optically pump only one single nanowire in our optical setup that can focus the pumping laser into a ~10 µm diameter spot. Fig. 3b presents the SEM image of a single transferred nanowire with a length of ~18 µm. A simulated cross-sectional optical mode profile of the transferred nanowire is shown in Fig. 3c. We confirmed via FDTD simulations that a single transferred nanowire can achieve a high optical quality (Q) factor of up to ~200 since the large refractive index contrast between the GeSn nanowire and the SiO_2_ layer prevents the optical field from leaking into the substrate (see Supplementary Note 3 for more details on the FDTD simulation). Fig. 3d presents the emission spectra from a single nanowire shown in Fig. 3b. All measurements were performed at 4 K. At a low pump power density of 96.6 kW cm^−2^, the spectrum exhibits a broadband spontaneous emission without any clear cavity resonances. At an increased pump power density of 120.7 kW cm^−2^, two cavity resonances are observed at ~2251 and ~2275 nm, respectively, which contrasts with only a broad spontaneous emission spectrum from the as-grown nanowires at a similar pumping level. At higher pump power densities of 181.1 and 301.8 kW cm^−2^, the two cavity modes become smaller in intensity and disappear, respectively. Fig. 3e shows a zoomed-in spectrum in a black dashed rectangle in Fig. 3d, which highlights the two cavity resonances with a free spectral range (FSR) of ~24 nm and a narrow full-width at half-maximum (FWHM) of ~3.3 nm for a peak at 2275 nm. We confirmed via FDTD simulations that the two cavity resonances are from the longitudinal modes in GeSn nanowire cavity (see Supplementary Note 4 for more details on the comparison study between simulated and experimental cavity resonances). We also studied the cavity linewidth change as a function of the pump power density (Inset to Fig. 3e). Since the cavity linewidth is directly related to the Q factor in the nanowire governed by the material’s loss and gain^36^, investigation of the pump-dependent linewidth provides a deeper understanding towards achieving lasing. At low pump power densities of 96.6 and 120.7 kW cm^−2^, the linewidth of the cavity mode at 2275 nm reduces from ~5.8 nm to ~3.3 nm, indicating an increased net gain as the pump power is increased. As the pump power density is further increased to 181.1 kW cm^−2^, the linewidth broadens to ~3.8 nm, showing the reduction in the net gain at the higher pump power. This change in optical net gain as a function of the pump power density will also be explained in the following net gain modeling section. It is worth noting that the emission from a transferred single nanowire is strongly polarized while the emission from as-grown nanowires does not show any polarization dependence (Supplementary Fig. 7 for more details)^37,38^.

### Theoretical modeling for gain and loss in single Ge/GeSn core/shell nanowire

**Fig. 4:**
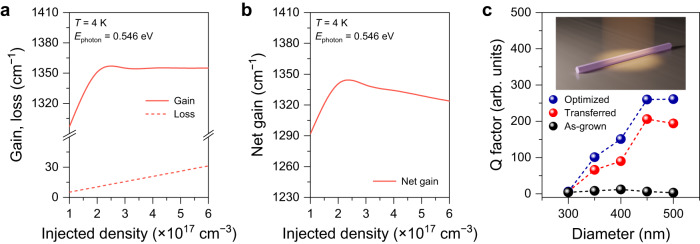
Theoretical modeling for gain and loss in single Ge/GeSn core/shell nanowire at 4 K. **a** Calculated gain (solid line) and loss (dashed line) for GeSn with an Sn content of 10.5 at% at the photon energy of 0.546 eV that corresponds to a wavelength of 2275 nm as a function of injected carrier density. The gain grows initially and reaches saturation at an injection density of ~2.2 × 10^17^ cm^−3^. The loss increases continuously as the injection density increases from 1 to 6 × 10^17^ cm^−3^. **b** Calculated net gain as a function of the injected carrier density. The peak of the net gain is at ~2.2 × 10^17^ cm^−3^, which corresponds to a pump power density of ~120 kW cm^−2^, at which the cavity modes are the strongest and narrowest. **c** Simulated Q factors at different nanowire diameters for an as-grown nanowire (black), a transferred nanowire (red), and an optimized nanowire (blue). At all simulated diameters, the Q factors of the transferred nanowire are >2 times greater than those of as-grown nanowires. The Q factors of the nanowire with an optimized structure are further improved by 30% as compared to the transferred nanowires at all simulated diameters. Inset: an optimized nanowire cavity structure used in the FDTD simulations.

For a comprehensive understanding of the changes in the intensity and linewidth of cavity modes, we performed theoretical modeling for the gain and loss dynamics in the GeSn nanowire (see Supplementary Note 5 for more details on the theoretical modeling). Fig. 4a shows the calculated material gain plotted with reverse sign (solid line) and loss (dashed line) as a function of injected carrier density at 4 K. The material gain increases initially and becomes saturated at an injection density of ~2.2 × 10^17^ cm^−3^, while the material loss increases steadily at the injection density range from 1 to 6 × 10^17^ cm^−3^. The material loss considers all possible loss mechanisms including free-carrier absorption (FCA) and inter-valence band absorption (IVBA) (see Supplementary Note 5 for more details). Fig. 4b shows the net gain that is computed by subtracting the loss from the gain. The net gain increases initially and starts decreasing at the injection density of ~2.2 × 10^17^ cm^−3^ because the gain saturates at that injection density while the loss keeps increasing. It is worth mentioning that the injection density of ~2.2 × 10^17^ cm^−3^ showing the peak net gain corresponds to the pump power density of ~120 kW cm^−2^, at which the experimentally measured cavity resonances are the strongest and narrowest (blue curve of Fig. 3d). This net gain trend as a function of the injection density shown in Fig. 4b clearly explains the experimental observation of the cavity resonances at different pump powers. When the net gain is the largest at the injection density of ~2.2 × 10^17^ cm^−3^, the measured single nanowire reaches close to the transparency regime, thus resulting in the emergence of cavity resonances.

To realize lasing in a single GeSn nanowire, the net gain should overcome the cavity loss^12^. This can be achieved by introducing tensile strain that can improve the net gain^39^ and also by improving the optical cavity. To investigate the possibility of improving the optical cavity of a single GeSn nanowire, we performed comprehensive FDTD simulations to study the *Q* factors of various nanowires. Fig. 4c shows the calculated Q factors as a function of the nanowire diameter for structures mimicking an as-grown nanowire (black dots) and a transferred single nanowire (unoptimized, red dots).

The Q factors of the transferred nanowire are >2 times higher than those of as-grown nanowires at all simulated diameters. These simulation results clearly present the superior optical confinement of the nanowire transferred onto an SiO_2_ layer compared to that of as-grown nanowires, thus explaining the experimental observation of cavity modes only from the transferred nanowire. In addition, it is evident from the simulation that it is important to have a large diameter of the nanowire for achieving high Q factors. By increasing the nanowire diameter, the effective refractive index increases^7^, and thus the refractive index contrast between the nanowire and the substrate can be substantially increased. This reduces the optical field leaking from the nanowire to the substrate, thereby increasing the optical confinement. The diameter of the single transferred nanowire that showed cavity resonances experimentally is ~500 nm, which can achieve good optical confinement according to the simulation.

We also designed an optimized nanowire structure as shown in the inset of Fig. 4c and calculated its Q factors (optimized, blue dots) to provide insight toward realizing a GeSn nanowire laser. The unoptimized structure has a flat facet on one side and a non-flat facet with a gold seed attached to it on another facet^18^, which scatters the photons and reduces the Q factor. We designed the optimized structure to have flat facets on both sides without a gold seed. This optimized structure can be achieved through a one-step aligned photolithography step. The optimized structure shows ~30% improved Q factors compared to the unoptimized nanowire at all simulated diameters because the optimized structure does not have the scattering site at a non-flat facet. We believe that it may be feasible to achieve lasing in the optimized structure, possibly also by harnessing tensile strain to improve the material gain since the observed cavity resonances in our measurements already show a very narrow FWHM of ~3 nm that is generally seen just before the lasing occurs^13,14,40^.

## Discussion

We have presented the experimental observation of cavity resonances in a single Ge/GeSn core/shell nanowire grown by bottom-up synthesis. The single nanowire transferred onto an oxidized Si substrate secures a large refractive index contrast between the nanowire and the SiO_2_ layer, which played a major role in enabling the observation of strong cavity modes in the nanowire. The experimentally measured spectra showing two strong cavity peaks are in excellent agreement with the FDTD simulation results, which confirm that the cavity resonances are due to the longitudinal modes confined in the GeSn nanowire. Theoretical modeling shows that the net gain increases initially and decreases at higher pump powers, which explains the behavior of the strong cavity resonances in our material system. The use of a thin Ge core in our study allowed for achieving a very uniform Sn content and a significantly lower compressive strain in the GeSn shell, which improves the nanowire optical properties thus paving the way to the observation of strong optical cavity modes. The compressive strain can be further relaxed by harnessing a highly selective Ge etching that can only remove the Ge core (see Supplementary Fig. 5). We have also shown the possibility of improving the nanowire optical cavity that can help achieve lasing in the GeSn nanowire. The deposition of an external stressor layer such as silicon nitride can induce tensile strain in the nanowire^41^ and improve the net gain^39^, thus leading to the realization of the first group-IV bottom-up nanowire laser. This experimental observation of cavity resonances in a single GeSn nanowire paves the way toward the realization of a group-IV nanowire laser, opening new possibilities for ultrasmall on-chip light sources in the SWIR range and beyond.

## Methods

### Nanowire growth

The Ge/Ge_0.90_Sn_0.10_ core/shell nanowires were grown in a chemical vapor deposition (CVD) reactor using germane (GeH_4_) and tin-tetrachloride (SnCl_4_) as precursor gases. Prior to the growth, the Ge(111) substrate was cleaned with a 2% HF solution and then the 20 nm Au colloids were deposited on the wafer, dried with Nitrogen flow, and then loaded in the CVD reactor. The vapor-liquid-solid (VLS) growth of the 20 nm Ge nanowires was performed for 20 min at 340 °C, while the Ge_0.90_Sn_0.10_ shell was grown for 150 min at 310 °C (Ge/Sn ratio in a gas phase of 2200). The sample for APT measurements was grown with identical parameters, but with a reduced shell growth time of 30 min.

### Scanning transmission electron microscopy

In order to carry out STEM, GeSn nanowires were swiped over a silicon piece to transfer them. The transferred wires were embedded using electron beam-deposited Carbon and Platinum as well as ion beam-induced platinum and then embedded in a TEM lamella using Focused Ion beam milling as described in [10.1557/mrs2007.63]. Focused Ion beam preparation was carried out in an FEI Helios Nanolab 660 DualBeam using a Ga ion beam at voltages between 30 and 5 kV. TEM imaging and EDX analysis were carried out in a Thermo Scientific Talos F200X G2 with ChemiSTEM technology at a voltage of 200 kV. The EDX data were analyzed using the Velox Software package.

### Atom probe tomography

Samples for APT were prepared by transferring single nanowires onto isolated tips in an FEI Helios Nanolab 660 DualBeam as described in [10.1021/acs.nanolett.6b03109]. APT was carried out in a Cameca LEAP 5000XS at a base temperature of 30 K, a detection rate of 4%, and a laser pulse energy of 5pJ. The APT data were reconstructed and analyzed using the IVAS software package.

### Photoluminescence spectroscopy

The samples were mounted into a closed-cycle helium cryostat (Cryostation s50, Montana Instrument) and measured at 4 K. A 1550 nm laser with a pulse width of 10 ns and repetition rate of 1 MHz was focused on the samples using a 15× reflective objective lens. The spot size was set to ~10 μm. For the as-grown nanowire sample, about 10 nanowires were optically pumped within the pumping spot. For the transferred nanowire sample, only a single nanowire with a length of ~18 μm was illuminated within the pumping beam area. The emitted signal from the samples was collected by the same reflective objective lens. The collected signal was then coupled into Fourier transform infrared (FTIR) interferometer and detected by InGaAs and InSb detectors.

### Reporting summary

Further information on research design is available in the Nature Portfolio Reporting Summary linked to this article.

## Supplementary information

Supplementary Information

Reporting Summary

## Data Availability

The data that support the findings of this study are available within the main text and Supplementary Information. Any other relevant data are available from the corresponding authors upon request.
