# Supplementary material for: Short-wave infrared cavity resonances in a single GeSn nanowire
Source: Nat Commun. 2023 Jul 20;14:4393. doi: 10.1038/s41467-023-40140-0 (PMC10359335; doi:10.1038/s41467-023-40140-0)
Supplement: Supplementary file 1 — Supplementary Information [file 41467_2023_40140_MOESM1_ESM.pdf]

Supplementary Information

Short-wave infrared cavity resonances in a single GeSn nanowire

Youngmin Kim[1†], Simone Assali[2†], Hyo-Jun Joo[1], Sebastian Koelling[2], Melvina Chen[1], Lu Luo[2], Xuncheng Shi[1], Daniel Burt[1], Zoran Ikonic[3], Donguk Nam[1*] and Oussama Moutanabbir[2*]

[1]School of Electrical and Electronic Engineering, Nanyang Technological University, 50 Nanyang Avenue, Singapore 639798, Singapore
[2]Department of Engineering Physics, École Polytechnique de Montréal, C.P. 6079, Succ. Centre-Ville, Montréal, Québec H3C 3A7, Canada
[3]School of Electronic and Electrical Engineering, University of Leeds, Leeds LS2 9JT, UK

[†]These authors contributed equally to this work.
[*]E-mail: dnam@ntu.edu.sg, oussama.moutanabbir@polymtl.ca

Table of Content

Note 1. Nanowire transfer procedure

Note 2. Raman analysis on nanowires before and after transfer process

Note 3. Optical mode confinement of as-grown and transferred nanowires

Note 4. Comparison between simulated and experimental cavity resonances

Note 5. Theoretical modeling of the gain and loss dynamics

Note 6. Atom probe tomography showing Sn radial profiles for Ge/GeSn core/shell nanowires grown using for thin and thick Ge core

Note 7. Ge core etching to release compressive strain in the GeSn shell

Note 8. Theoretically calculated bandgap energies for Γ- and L-valleys as a function of Sn content at 4 K

Note 9. Polarization dependence of the emission from as-grown nanowires and a transferred single nanowire

References

Note 1. Nanowire transfer procedure

To transfer the as-grown nanowires onto the SiO_2 layer, we prepared an as-grown nanowire sample and an Si substrate coated with a thermally grown SiO_2 layer. We rubbed the as-grown nanowires directly against the oxidized Si substrate. The as-grown nanowires were then transferred onto the SiO_2 layer. During the transfer process, the nanowires were evenly distributed from low density to high density on the entire SiO2 layer. We investigated an area where nanowires area sparsely distributed through an optical microscope and conducted experiments in the area where only one single nanowire can be optically pumped.

Note 2. Raman analysis on nanowires before and after transfer process

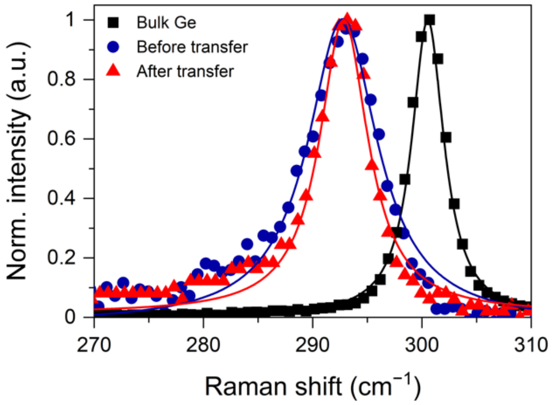

Supplementary Figure 1 | Raman characterization of as-grown and transferred nanowire samples. Raman spectra of as-grown (blue curve) and transferred nanowire (red curve) samples showing peak positions of 292.7 and 292.8 cm^{-1}, respectively. A Raman spectrum of a bulk Ge (black curve) with a peak position of 300.5 cm^{-1} is provided for reference. The single transferred nanowire shows a narrower FWHM of ~5.41 cm^{-1} than that of as-grown nanowires (~7.79 cm^{-1}), indicating multiple nanowires being optically pumped simultaneously in the as-grown sample. The corresponding Sn content from the Raman peak position is calculated to be ~10 at.%.

To confirm that the transfer process does not induce any damage to the nanowires, we conducted the Raman spectroscopy measurement, which can assess defects and disorders in materials[1]. Supplementary Figure 1 shows the measured Raman spectra of the as-grown vertically standing nanowires (blue curve) and a single transferred nanowire (red curve). A spectrum from bulk Ge (black curve) is also included as a reference. We confirmed that while the peak positions of the two samples are nearly consistent, the single transferred nanowire shows a narrower full-width at half-maximum (FWHM) of ~5.41 cm^{-1} than that of as-grown vertical nanowires (~7.79 cm^{-1}). For as-grown vertical nanowires, approximately 3 nanowires are simultaneously excited by the 532-nm pump laser with a focused laser spot size of ~1 μm. As each nanowire may have slightly different physical characteristics (e.g., Sn content, residual stress, physical dimension, etc.), the measured Raman spectra from multiple vertical nanowires can display a larger FWHM compared to a single transferred nanowire. From the Raman peak position, we can deduce an Sn content of ~10 at%, which is consistent with the Atom Probe Tomography (APT) result.

Note 3. Optical mode confinement of as-grown and transferred nanowires

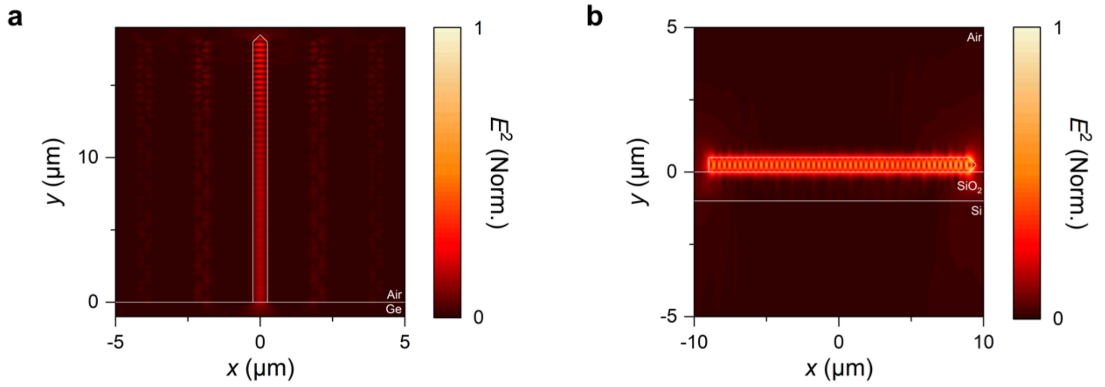

Supplementary Figure 2 | Simulated optical mode profiles of as-grown and transferred nanowires. a Cross-sectional mode profile of an as-grown nanowire, showing weak optical fields due to significant leakage of fields from the GeSn nanowire to the Ge substrate. **b** Cross-sectional mode profile of a transferred nanowire, showing strong optical confinement due to large index contrast between a GeSn nanowire and an SiO_2 layer.

To investigate whether a transferred nanowire could confine the light better than an as-grown nanowire, we performed finite-difference time-domain (FDTD) optical simulations. The simulated wavelength is set to 2275 nm which is the peak wavelength of our resonance spectrum. GeSn (480 nm), Ge (20 nm) and Si with refractive indices of 4.3[2], 4.1 and 3.4, respectively, were employed for the as-grown nanowire simulation. For the transferred nanowire simulation, an SiO_2 with a refractive index of 1.44 is additionally employed above the Si substrate. Supplementary Figure 2a shows a simulated cross-sectional optical mode profile of the vertically standing as-grown nanowire. As shown in the Ge layer, there is a significant leakage of optical fields from the GeSn nanowire to the Ge substrate, thereby showing weak optical fields in the nanowire. The small refractive index contrast between the nanowire and the substrate causes the leakage of fields, hindering optical confinement in the nanowire. In stark contrast to a poor optical confinement in the as-grown nanowire, a nanowire transferred onto an SiO_2 layer shows strong optical confinement as shown in Supplementary Fig. 3b. The large refractive index difference between the GeSn nanowire and the SiO_2 layer prevents the leakage of fields from the nanowire to the Si substrate, enabling strong optical confinement in the nanowire.

Note 4. Comparison between simulated and experimental cavity resonances

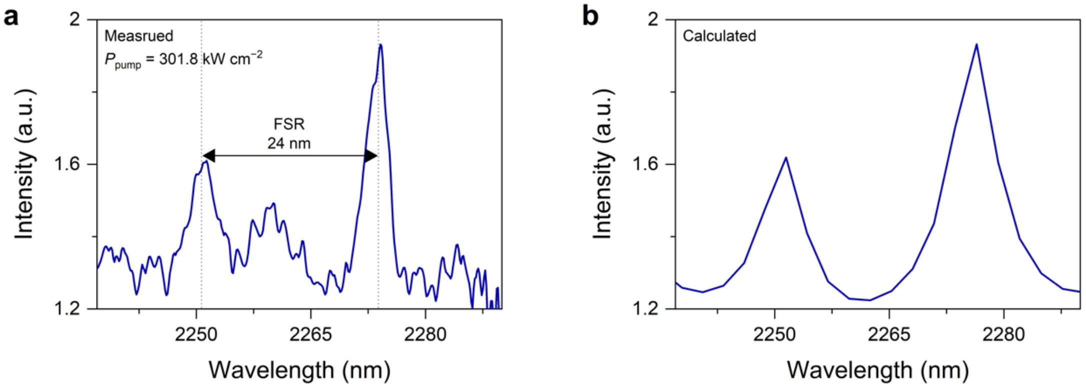

Supplementary Figure 3 | Simulated and measured spectra of transferred nanowire. a Magnified view of a spectrum experimentally measured at 120.7 kW cm^{-2} (black dashed line in Fig. 3d), showing two strong cavity resonances with an FSR of ~24 nm. **b** Theoretical spectrum calculated by FDTD optical simulation, exhibiting a mode spacing of ~25 nm. The experimental value corresponds to the calculated value, confirming that resonances are attributed to the longitudinal cavity in the nanowire.

To study whether the cavity resonances originate from the longitudinal modes in GeSn nanowire cavity, we compared the observed free spectral range (FSR) with a theoretical mode spacing calculated by FDTD optical simulation. Supplementary Figure 3a presents a magnified view of two strong cavity modes taken at 120.7 kW cm^{-2} (black dashed line in Fig. 3d), exhibiting an FSR of ~24 nm. Supplementary Figure 3b shows two modes with a space of ~25 nm calculated by FDTD optical simulation. A simulated spectrum using FDTD simulation that is in excellent agreement with the experimental observation. The theoretically calculated value (~25 nm) corresponds to the experimentally measured value (~24 nm), thus confirming that resonances stem from the longitudinal cavity in the nanowire.

Note 5. Theoretical modeling of the gain and loss dynamics

The interband gain, and also the inter-valence band absorption, were calculated from the 8-band **k·p** model, using the expressions from Chuang *et al.*[3]. The Luttinger parameters of GeSn alloy are calculated according to Liu *et al.*[4], and other parameters are listed in Rainko *et al.*[5]. To find the net gain, the free-carrier absorption can be calculated using the second order perturbation model described in Tsai *et al.*[6], with the acoustic phonon, deformation potential (L-valley), intervalley, ionized impurity, and alloy scattering included. Alternatively, it can be calculated from expressions coming from fitting to experiment for Ge[7,8], and we have here used the later option. Finally, the indirect absorption (towards the L- and X-valleys in conduction band) which may be important in group-IV materials, was also included, as described in Virgilio *et al.* and Trupke *et al.*[9,10], but in this particular system it turned out to be negligible.

Note 6. Atom probe tomography showing Sn radial profiles for Ge/GeSn core/shell nanowires grown using for thin and thick Ge core

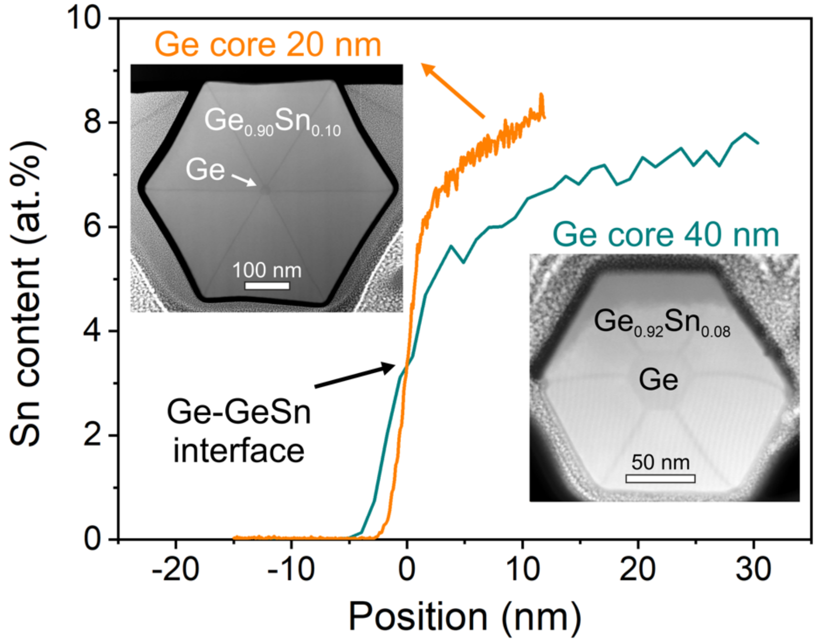

Supplementary Figure 4 | Atom probe tomography (APT) showing Sn radial profiles for Ge/GeSn core/shell nanowires grown using thin (orange) and thick (dark cyan) Ge core. Inset: Representative cross-sectional transmission electron microscope (TEM) images of these two nanowires. Note that the growth on thin Ge core is associated with a faster increase in Sn content.

To further highlight the role of the core diameter, Supplementary Figure 4 compares the Sn radial profiles measured by atom probe tomography content close to Ge/GeSn interface for a nanowire with a thin core and another with a thick core. We note that for a thin core the Sn content increases rapidly as the GeSn shell growth progresses leading to higher and uniform radial composition (Fig 1. d) of the manuscript. Moreover, this protocol can be straightforwardly extended to control the Sn content in the Ge/GeSn core/shell nanowires by adjusting the growth temperature. This achieved uniformity of the Sn content paved the way to clearly observe the cavity modes by allowing decent gain to be obtained in nanowires, as reported in the manuscript.

Note 7. Ge core etching to release compressive strain in the GeSn shell

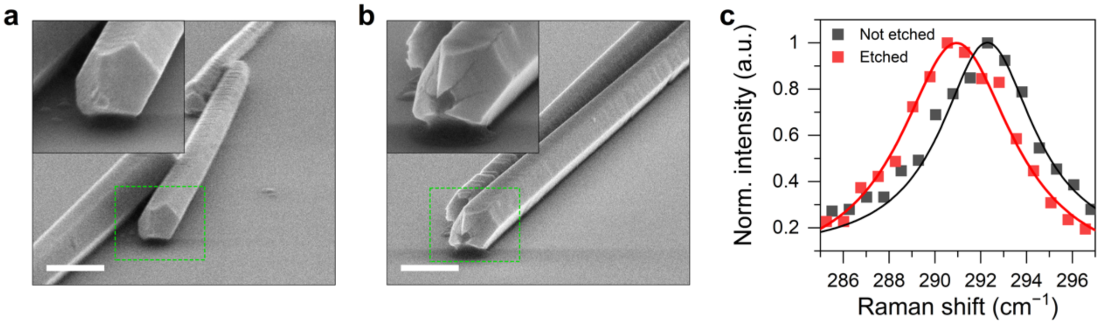

Supplementary Figure 5 | Ge core etching to release compressive strain in the GeSn shell. a Tilted-view SEM image before etching the Ge core. **b** Tilted-view SEM image after etching the Ge core, showing a hollow shape. **c** Raman spectra of the single transferred nanowire before (black curve) and after (red curve) the core etching, showing peak positions of 292.3 and 290.9 cm^{-1}, respectively.

To provide the experimental evidence of the small compressive strain in the GeSn shell, we conducted additional experiments involving the selective removal of the Ge core that induces the compressive strain. By exploiting the Ge selective etching technique into our nanowire[11], we successfully etched the Ge core and performed Raman spectroscopy to measure how much the strain is relaxed in the GeSn shell. Supplementary Figures 5a and 5b present SEM images of the single transferred nanowire before and after etching process. The post-etch SEM image shows a hollow core after etching the Ge core. We conducted Raman spectroscopy to evaluate the compressive strain before and after the core etch. Supplementary Figure 5c shows the measured Raman spectra of the single transferred nanowire before (black curve) and after (red curve) etching. The peak positions of nanowires before and after etching are centered at ~292.3 and ~290.9 cm^{-1}, respectively. This indicates the relaxation of compressive strain in the GeSn shell after the etching process. The peak shift of 1.4 cm^{-1} corresponds to a compressive strain of about −0.27%, confirming that the GeSn shell has a very small compressive strain, which is enabled by using very thin Ge core as nanoscale substrate. It should be noted that the previous studies using a thicker Ge core of ~50 nm reported a compressive strain of ~−0.7%, which is significantly larger than the value in this study.

Note 8. Theoretically calculated bandgap energies for Γ- and L-valleys as a function of Sn content at 4 K

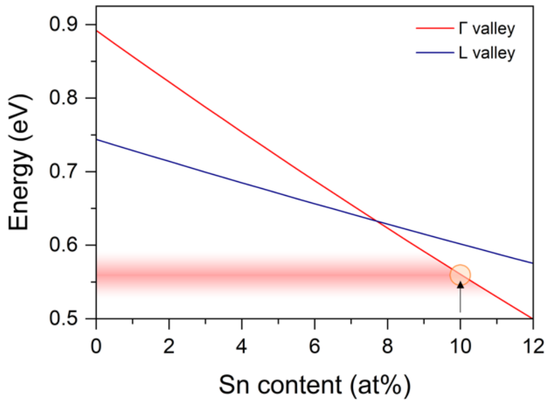

Supplementary Figure 6 | Theoretically calculated bandgap energies for Γ- and L-valleys as a function of Sn content at 4 K. The theoretically calculated bandgap energy of 0.5505 eV for GeSn with a 10 at% Sn content is highlighted in an orange circle. The red gradient area indicates the experimentally measured emission bandwidth.

To further corroborate the direct bandgap nature of our GeSn nanowire, we additionally employed **k·p** theory to calculate bandgap energies of Γ- and L-valleys as a function of Sn content and compared the theoretically calculated results with our experimental results. Supplementary Figure 6 shows calculated bandgap energies of Γ- and L-valleys as a function of Sn content at 4 K, showing that the indirect-to-direct cross-over occurs at a Sn content of ~8 at%. The Sn content of 10 at% in our nanowire, which is an experimentally measured value via Atom Probe Tomography, and a reduced compressive strain of −0.27%. This content and strain level are beyond this indirect/direct cross-over point. Indeed, the calculated bandgap energy for the Γ valley is 0.5505 eV, which corresponds to a wavelength of 2252 nm, which is in excellent agreement with the experimentally measured emission peak of 2251 nm (highlighted in an orange circle in Supplementary Figure 6). This theoretical benchmarking further confirms the direct bandgap nature of our GeSn nanowire in addition to the temperature-dependent photoluminescence characteristics.

Note 9. Polarization dependence of the emission from as-grown nanowires and a transferred single nanowire

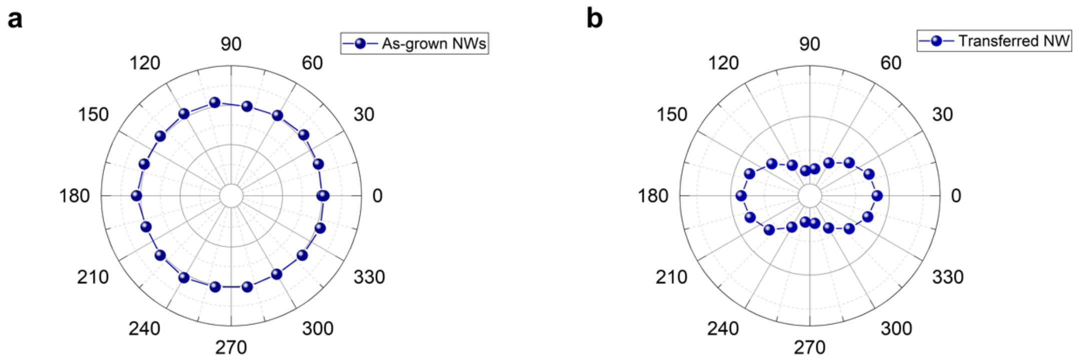

Supplementary Figure 7 | Polarization dependence of the emission from as-grown nanowires and a transferred single nanowire. a Polarization dependence of the emission from as-grown nanowires. **b** Polarization dependence of the emission from a transferred single nanowire. It is shown that the emission from a transferred single nanowire is highly polarized along the longitudinal axis of the nanowire due to the optical mode confinement in the single nanowire.

To confirm the effect of polarization angle of detection, we first measured the polarization dependence of the emission from as-grown nanowires and a single nanowire by placing a linear polarizer in front of the detector. Supplementary Figure 7a and 7b show the integrated photoluminescence intensity as a function of detection polarization angle for as-grown nanowires and a single nanowire, respectively. The angle is defined as the relative angle between the linear polarizer and a longitudinal axis of the transferred nanowire. While the as-grown nanowires do not show polarization dependence, the emission from the transferred single nanowire is polarized along the longitudinal axis of the nanowire. This polarized emission is due to the optical mode confinement in the single nanowire, which is consistent with the literature[12]. The excitation polarization dependence for both as-grown nanowires and transferred nanowires were found to be the same as the detection polarization dependence, showing unpolarized and polarized emissions, respectively, which are also consistent with the literature[13].

References

1. Gouadec, G. & Colomban, P. Raman Spectroscopy of nanomaterials: How spectra relate to disorder, particle size and mechanical properties. *Prog. Cryst. Growth Charact. Mater.* **53**, 1–56 (2007).
2. Tran, H. *et al.* Systematic study of Ge $_{1-x}$ Sn $_x$ absorption coefficient and refractive index for the device applications of Si-based optoelectronics. *J. Appl. Phys.* **119**, 103106 (2016).
3. Chaung, S. L. *Physics of photonic devices*. (Wiley New York, 2009).
4. Liu, S.-Q. & Yen, S.-T. Extraction of eight-band k · p parameters from empirical pseudopotentials for GeSn. *J. Appl. Phys.* **125**, 245701 (2019).
5. Rainko, D. *et al.* Investigation of carrier confinement in direct bandgap GeSn/SiGeSn 2D and 0D heterostructures. *Sci. Rep.* **8**, 15557 (2018).
6. Chin-Yi Tsai *et al.* Theoretical model for intravalley and intervalley free-carrier absorption in semiconductor lasers: beyond the classical Drude model. *IEEE J. Quantum Electron.* **34**, 552–559 (1998).
7. Liu, J. *et al.* Tensile-strained, n-type Ge as a gain medium for monolithic laser integration on Si. *Opt. Express* **15**, 11272 (2007).
8. Peschka, D. *et al.* Modeling of Edge-Emitting Lasers Based on Tensile Strained Germanium Microstrips. *IEEE Photonics J.* **7**, 1–15 (2015).
9. Virgilio, M., Manganelli, C. L., Grosso, G., Pizzi, G. & Capellini, G. Radiative recombination and optical gain spectra in biaxially strained n -type germanium. *Phys. Rev. B* **87**, 235313 (2013).
10. Trupke, T., Green, M. A. & Würfel, P. Optical gain in materials with indirect transitions.

J. Appl. Phys. **93**, 9058–9061 (2003).

11. Gupta, S. *et al.* Highly Selective Dry Etching of Germanium over Germanium–Tin ($Ge_{1-x}Sn_x$): A Novel Route for $Ge_{1-x}Sn_x$ Nanostructure Fabrication. *Nano Lett.* **13**, 3783–3790 (2013).
12. Tatebayashi, J. *et al.* Room-temperature lasing in a single nanowire with quantum dots. *Nat. Photonics* **9**, 501–505 (2015).
13. Wang, J., Gudiksen, M. S., Duan, X., Cui, Y. & Lieber, C. M. Highly Polarized Photoluminescence and Photodetection from Single Indium Phosphide Nanowires. *Science* **293**, 1455–1457 (2001).
